# Monitoring health systems readiness and inpatient malaria case-management at Kenyan county hospitals

**DOI:** 10.1186/s12936-018-2364-8

**Published:** 2018-05-29

**Authors:** Dejan Zurovac, Beatrice Machini, Rebecca Kiptui, Dorothy Memusi, Beatrice Amboko, Samuel Kigen, Patricia Njiri, Ejersa Waqo

**Affiliations:** 1 KEMRI-Wellcome Trust Research Programme, Nairobi, Kenya; 2 Centre for Tropical Medicine and Global Health, University of Oxford, Oxford, UK; 3 National Malaria Control Programme, Ministry of Health, Nairobi, Kenya; 4 Clinton Health Access Initiative, Nairobi, Kenya

**Keywords:** Artesunate, Adherence, Guidelines, Health systems, Case-management

## Abstract

**Background:**

Change of severe malaria treatment policy from quinine to artesunate, a major malaria control advance in Africa, is compromised by scarce data to monitor policy translation into practice. In Kenya, hospital surveys were implemented to monitor health systems readiness and inpatient malaria case-management.

**Methods:**

All 47 county referral hospitals were surveyed in February and October 2016. Data collection included hospital assessments, interviews with inpatient health workers and retrospective review of patients’ admission files. Analysis included 185 and 182 health workers, and 1162 and 1224 patients admitted with suspected malaria, respectively, in all 47 hospitals. Cluster-adjusted comparisons of the performance indicators with exploratory stratifications were performed.

**Results:**

Malaria microscopy was universal during both surveys. Artesunate availability increased (63.8–85.1%), while retrospective stock-outs declined (46.8–19.2%). No significant changes were observed in the coverage of artesunate trained (42.2% vs 40.7%) and supervised health workers (8.7% vs 12.8%). The knowledge about treatment policy improved (73.5–85.7%; p = 0.002) while correct artesunate dosing knowledge increased for patients < 20 kg (42.7–64.6%; p < 0.001) and > 20 kg (70.3–80.8%; p = 0.052). Most patients were tested on admission (88.6% vs 92.1%; p = 0.080) while repeated malaria testing was low (5.2% vs 8.1%; p = 0.034). Artesunate treatment for confirmed severe malaria patients significantly increased (69.9–78.7%; p = 0.030). No changes were observed in artemether–lumefantrine treatment for non-severe test positive patients (8.0% vs 8.8%; p = 0.796). Among test negative patients, increased adherence to test results was observed for non-severe (68.6–78.0%; p = 0.063) but not for severe patients (59.1–62.1%; p = 0.673). Overall quality of malaria case-management improved (48.6–56.3%; p = 0.004), both for children (54.1–61.5%; p = 0.019) and adults (43.0–51.0%; p = 0.041), and in both high (51.1–58.1%; p = 0.024) and low malaria risk areas (47.5–56.0%; p = 0.029).

**Conclusion:**

Most health systems and malaria case-management indicators improved during 2016. Gaps, often specific to different inpatient populations and risk areas, however remain and further programmatic interventions including close monitoring is needed to optimize policy translation.

## Background

After decades of quinine use for severe malaria treatment in Kenya, the National Malaria Control Programme (NMCP) launched in 2012 the new guidelines for the management of severe malaria [1] and recommended change of treatment policy to injectable artesunate—the treatment recommended by the World Health Organization (WHO) [2] and shown to reduce malaria mortality in multicentre trials, including those undertaken in Kenya [3, 4]. In the following years, the policy implementation was supported with procurement and distribution of artesunate alongside a series of in-service trainings for health workers on inpatient malaria management [5]. Despite the importance of inpatient care, routine logistic and health information systems provide scarce information about hospital and health worker readiness to implement malaria case-management policies and their actual practices in delivering inpatient care [6, 7]. Few studies across Africa have examined this topic and of published reports, various assessments were commonly limited to paediatric populations, rarely examined coverage with interventions, included small numbers of facilities, and if done on larger scale, were often not followed up to monitor trends of the policy implementation [8–16]. This has been in contrast with outpatient studies often reporting major improvements in the implementation of test and treat policy for malaria, both in Kenya [17] and in other African countries [18]. For instance, in Kenya between 2010 and 2016 overall adherence to outpatient test and treat guidelines increased from 16–59% [19]. With respect to inpatients, by the end of 2014, all of 47 Kenyan county referral hospitals were supplied with artesunate and in-service trainings for health workers reached all counties countrywide. Subsequently the NMCP launched biannual hospital surveys at county referral hospitals to monitor levels and trends in health systems readiness to implement new treatment policy; health workers’ coverage with interventions and their treatment knowledge; and malaria case-management practices for patients admitted to paediatric and medical wards. The main findings of the first two inpatient surveys undertaken in 2016 are reported.

## Methods

### Context and general study design

**Fig. 1:**
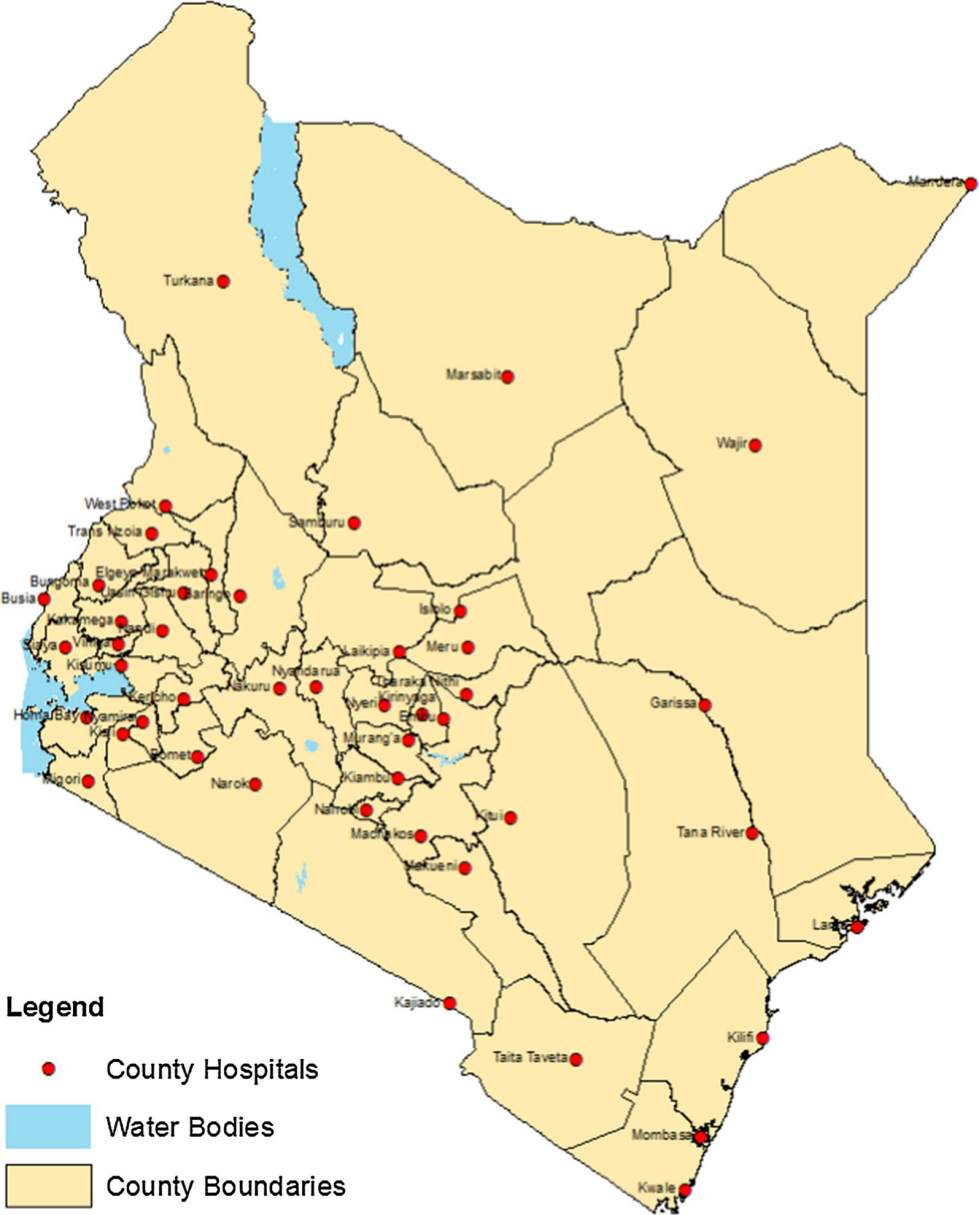
Map of county hospitals in Kenya

The general study design are biannual monitoring surveys including all 47 county referral hospitals (Fig. 1). All hospitals are public and government owned. The majority (37/47) are internship hospitals providing supervised practice to medical doctors and clinical officers for one year prior to full registration. The median number of paediatric and medical ward beds is 32 and 52 respectively and they range across hospitals from 6 to 74 beds for paediatric wards and from 26 to 106 beds for medical wards. Of 47 hospitals, 13 are located within high malaria risk counties in lake and coast endemic areas where prevalence of childhood malaria infection of 26.7 and 8.1% was respectively reported during the community survey in 2015 [20]. The remaining hospitals are within low risk areas with malaria prevalence ranging from 0.3 to 3.1% [20]. Of relevance for malaria case-management, all hospitals procure medicines from Kenya Medical Supply Agency and injectable artesunate is supplied for free through external subsidies. Since 2012, health workers have been exposed to two types of NMCP coordinated in-service trainings: (a) 3-day NMCP malaria case-management workshops, implemented annually for health workers through the counties and various training institutions following standard curriculum with half a day devoted to severe malaria [21]; and (b) artesunate focused trainings for hospital health workers implemented through one nationwide round of half a day continuous medical education (CME) sessions. In addition, some inpatient health workers were exposed to integrated 5-day paediatric trainings where half a day is devoted to the management of severe malaria. During malaria specific trainings, national malaria guidelines and artesunate administration job aids were distributed to all participants. Finally, either integrated or malaria specific supervision supported by the NMCP was ideally provided on quarterly basis by the County Health Management Teams.

### National recommendations, monitoring indicators and study definitions

Key monitoring indicators reflected recommendations from national malaria guidelines [1], training manuals [21] and artesunate administration guidelines [22]. Selection of indicators also considered those that can be extracted using retrospective review of medical files, interviews with health workers and hospital assessments. The composite case-management performance indicator referring to clinical practices among suspected malaria admissions was constructed of recommended malaria testing and treatment practices. The indicator reflects national guidelines specifying that *“in all patients with suspected severe malaria the use of parasitological diagnosis is recommended irrespective of whether the patient had fever or history of fever”* and that “*the recommended treatment for severe malaria is parenteral artesunate*”. The guidelines define severe malaria as detection of malaria parasitaemia and the presence of any of the following clinical and laboratory criteria: prostration (inability to drink, breastfeed, sit, stand, walk); alteration of consciousness level (from drowsiness to coma); respiratory distress (acidotic breathing); convulsions (2 or more); shock; pulmonary oedema; abnormal bleeding; jaundice; haemoglobinuria; acute renal failure (oliguria or anuria); severe anaemia (Hb < 5 g/dl or HCT < 15%); hypoglycaemia (blood glucose < 2.2 mmol/l) and hyperlactataemia. With respect to the treatment of test negative patients with severe malaria features the guidelines and training materials are not explicit that anti-malarial treatment should not be given but do promote repeated malaria testing and treatment discontinuation for negative results. Anti-malarial treatment of test negative patients without severe malaria criteria is unambiguously not recommended.

**Fig. 2:**
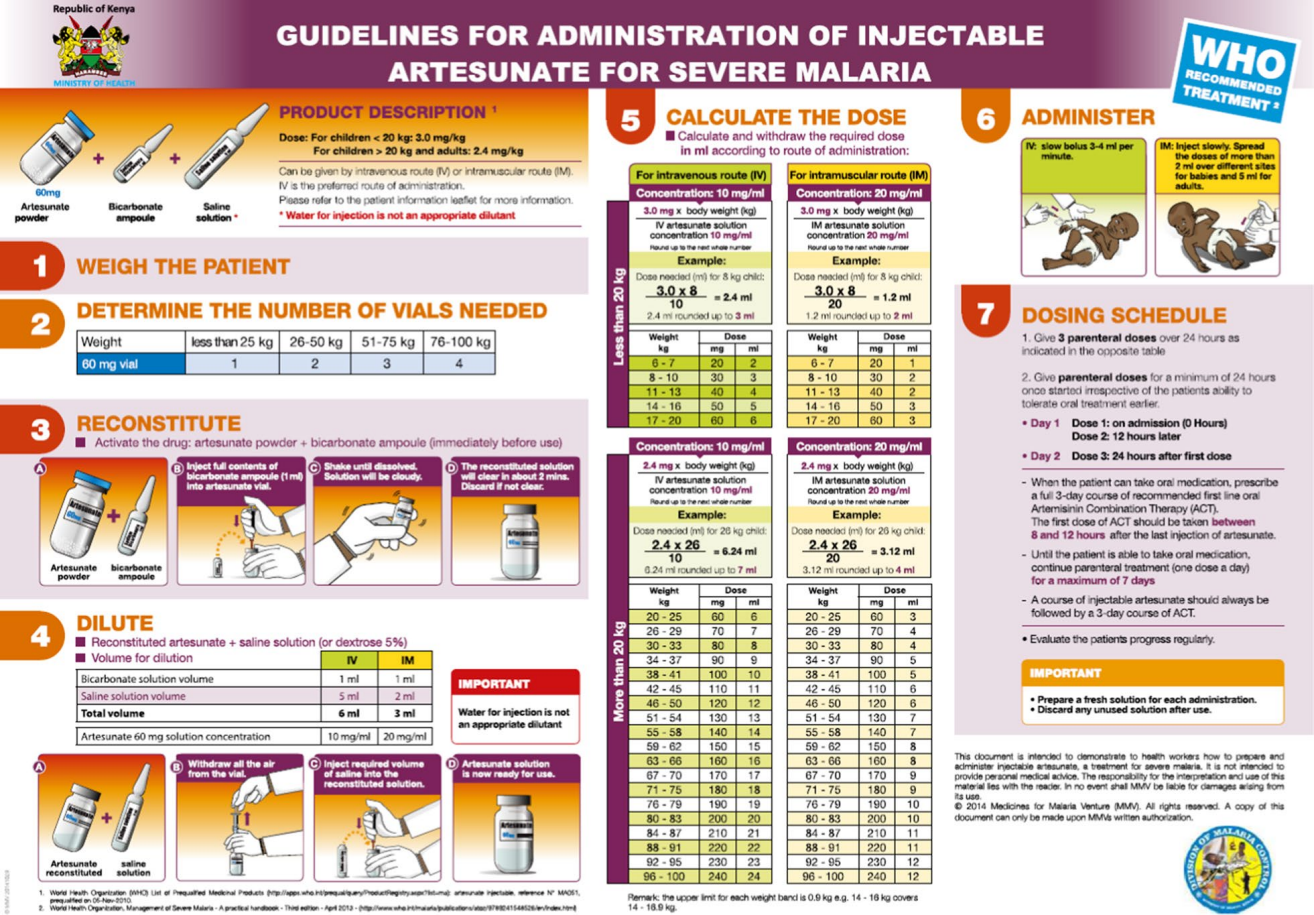
National guidelines for artesunate administration [22]

Therefore, to evaluate the quality of malaria case-management for patients admitted with suspected malaria, the composite case-management indicator was defined as performance of all of the following tasks: (1) testing of suspected malaria patients, and (2) prescribing of recommended treatment based on the severity criteria and malaria test results defined as (a) parenteral artesunate for severe test positive patients (confirmed severe malaria), (b) artemether–lumefantrine (AL) for non-severe test positive patients, (c) no anti-malarial treatment for severe test negative patients (or artesunate treatment with repeated malaria test followed by treatment discontinuation for negative results), and (d) no anti-malarial treatment for non-severe test negative patients. Recognizing that documentation of clinical criteria may not be optimal and to protect correctness of health workers’ treatment practices from documentation biases, the clinical severity criteria were complemented with health workers’ diagnosis of severe malaria made on admission. The other indicators at the patient level address performance of the testing and treatment practices for each of the individual components of the composite case-management. The key indicators at health facility and health worker level refer to the coverage of hospitals and inpatient health workers with interventions relevant for the management of severe malaria such as the availability of anti-malarials, malaria diagnostics and microscopy support, and exposure to relevant in-service trainings, guidelines and supportive supervision. Finally, the knowledge of health workers about treatment policy for severe malaria and artesunate use was measured in comparison with recommendations (Fig. 2).

### Sample size estimation

The sample size was calculated to detect statistically significant difference of at least 10% points around composite case-management indicator at the patient level between any two survey points during the monitoring period in each admission ward. The sample size was adjusted to take into consideration clustering effect due to homogeneity of practices at the hospital level. Therefore, in order to detect 10% difference around conservative estimates of 45% performance with the level of confidence of 5%, power of 80%, design effect of 1.8 [13, 14], a sample size of 704 suspected malaria admissions per ward (paediatric and adults) or 1408 patient files in total was estimated. To obtain estimated sample size from 47 county hospitals 15 patient files with suspected malaria admissions were to be extracted from each ward (704/47) or 30 files in total per hospital.

### Survey personnel, training and data collection procedures

The surveys were conducted with ten teams and each team was allocated 4–5 county hospitals. Each team comprised of three data collectors and was composed of one hospital medical records officer and two nurses of which at least one had extensive hospital experience. The teams were trained over 5 days in the week prior to data collection and the training included updates on severe malaria management; county and hospital introductions, survey procedures and patient files selection; theory of completing hospital assessments forms; theory and practice of data extraction based on real, anonymized medical files; theory and role play practice for health worker interviews and knowledge assessments; inter-team concordance testing for data collection tools; and practice of taking written informed consent.

At each of the hospitals data were collected over three consecutive days using three methods of data collection: (1) retrospective review of patients’ files from hospital medical records office; (2) interviews with paediatric and medical ward health workers and (3) hospital assessments. Prior to data extraction from patient files, the screening of the inpatient and laboratory registers was undertaken to select 30 consecutive files (15 from paediatric and 15 from medical wards) meeting criteria of suspected malaria defined as any form of malaria diagnosis made, malaria test performed or anti-malarial treatment prescribed. From each ward patients discharged in chronological order counting backwards prior to the survey day, up to a maximum of 6 months, were included. Thereafter, from each of 30 selected patient files, data elements were extracted from all available forms including structured and unstructured admission, continuation, observation, treatment, nursing cardex, discharge and laboratory forms, depending on the type of records used within the hospital. The main variables to be extracted included age, sex, weight, dates of admission and discharge, assessments and laboratory tests performed with results recorded, diagnoses made, and treatments prescribed. The presence of clinical criteria of severe malaria was established on admission as documented either at the casualty records or within 24 h upon admission to the ward. All patients having malaria test ordered and no result recorded in the file were traced back to the laboratory register to establish performance and result of the test.

The second data collection method included interviews with health workers. From each ward (paediatric and combined male and female medical wards) one nurse and one clinician on duty during the day shift of the first survey day were randomly selected for interviews. The information collected during interviews included health worker’s demographics, exposure to relevant in-service trainings, guidelines, supportive supervision, and their knowledge about management of severe malaria focusing on artesunate use. The knowledge was assessed using self-administered, multiple choice questions. Finally, the availability of commodities on survey day, the presence of stock-outs during 3 months preceding the survey, display of artesunate job aids, and basic equipment and services relevant for malaria case-management, were assessed in all hospitals at appropriate departments such as paediatric and medical wards, pharmacy and laboratory. During the interviews, all health workers were: (a) communicated correct responses for incorrectly responded knowledge questions; (b) provided national malaria case-management guidelines; and (c) delivered artesunate dosing job aids. In each of the study wards the presence of artesunate administration posters was assessed and, if absent, they were displayed by the survey teams. During the hospital stay informal conversations about malaria case-management and hospital readiness to support it were also held between study teams and ward clinicians and nurses, pharmacists and laboratory personnel.

### Data management and statistical analysis

Data entry, coding and management was undertaken using Access (Microsoft, USA), and thereafter analysed in STATA, version 13 (StataCorp, USA). Descriptive analyses were performed at the health facility, health worker, and patient levels. First, to assess health facility readiness to implement recommended malaria case-management the analysis was undertaken at the hospital level. Second, to assess health worker readiness for policy implementation, the analyses of health workers’ coverage with support interventions and their knowledge about new treatment policies, were performed at the health worker level. Third, to assess the quality of malaria case-management in accordance with national guidelines, the analysis was performed at the patient level. Correctness of case-management was analysed from the health workers’ malaria suspicion perspective, without considering comorbidities and focusing on anti-malarial test and treat practices. The primary analysis measured absolute percentage-point changes in the indicators between two surveys. The exploratory analyses, measuring percentage-point changes over time within strata and differences between strata, were also undertaken. The stratification at the hospital level was done by malaria risk (high vs low); at the health worker level by their cadre (clinicians vs nurses), exposure to the training (trained on artesunate use vs untrained) and ward allocation (paediatric vs combined male and female medical); and at the patient level by admission ward and malaria risk. To test statistical significance Chi square test for comparison of proportions was used. All p-values at patient and health worker levels were adjusted for clustering at the hospital level. Hypothesis testing were done with an alpha level of 0.05.

## Results

### Description of study populations

Surveys at 47 county hospitals were carried out between 8th and 26th February and between 26th September and 14th October 2016. During two surveys 185 and 182 inpatient health workers were interviewed. With respect to their pre-service training and ward allocation, the characteristics were similar between rounds with 49.7 and 51.1% being from paediatric ward, and 50.8 and 53.9% being nurses. Of the remaining 91 and 84 clinicians interviewed during the surveys, the majority were either clinical or medical officer interns (64.8% vs 65.5%). Conversely medical officers, clinical officers or consultants represented about a third of the clinicians, without differences between the surveys (35.1% vs 34.5%). In both rounds interviewed health workers had a median of 30 years of age and 4 years of inpatient experience.

During two survey rounds 1162 and 1224 suspected malaria admission files were reviewed of which respectively 1050 (90.4%) and 1084 (89.8%) met selection criteria based on any form of malaria admission diagnosis, 557 (47.9%) and 552 (49.1%) based on malaria testing, and 103 (8.9%) and 153 (12.9%) based on anti-malarial prescriptions, as recorded in the registers. Most patients, during both rounds, were discharged within 3 months prior to the surveys (77.0 and 66.8%). During both rounds 51% of reviewed files were for paediatric patients, male patients represented 53% of admissions, and median duration of illness prior to admission was 3 days. The median age of paediatric patients was 3 years at both rounds, while at the respective rounds the age of medical ward patients was 30 and 29 years. The length of patients’ admission was similar between rounds with 3 and 4 days respectively. Among reviewed files, the most common documented features of severe malaria included altered consciousness, prostration and repeated convulsions (Table 1). Additionally, severe anemia and respiratory distress were commonly documented among paediatric admissions. Overall, nearly half of the admission files had at least one documented feature of malaria severity on admission, the proportion slightly higher during the second round compared to the first round (49.8% vs 43.2%). During both rounds, health workers most commonly made unclassified diagnoses of “malaria” on admission (58.0 and 52.7%) while severe malaria diagnoses were made for nearly a third of patients (31.8 and 33.3%) (Table 1). Complementing clinical features with routine diagnoses, five categories of patients were determined to assess malaria case-management practices. During both rounds, the largest category comprised confirmed severe malaria patients (34.9 and 38.0%) defined as the presence of positive malaria test on admission, and either documentation of any features of severe malaria or severe malaria diagnosis made by a clinician. The remaining four categories included the following patient groups similarly distributed between survey rounds: (a) test positive non-severe malaria patients (18.2 and 18.6%); (b) test negative patients with severity criteria (17.9 and 19.6%); (c) test negative patients without severity criteria (17.6 and 15.9%) and (d) patients not tested for malaria regardless of the clinical features (11.4 and 7.9%).

**Table 1:** Documented severity criteria and malaria admission diagnoses, by ward and survey round

	Round 1			Round 2		
	Paediatric ward (N = 588)	Medical ward (N = 574)	All patients (N = 1162)	Paediatric ward (N = 620)	Medical ward (N = 604)	All patients (N = 1224)
	n (%)	n (%)	n (%)	n (%)	n (%)	n (%)
Clinical or laboratory features						
Altered consciousness^a^	77 (13.1)	124 (21.6)	201 (17.3)	108 (17.4)	147 (24.3)	255 (20.8)
Prostration^b^	79 (13.4)	38 (6.6)	117 (10.1)	125 (20.2)	51 (8.4)	175 (14.3)
Convulsions (2 or more)	95 (16.2)	15 (2.6)	110 (9.5)	108 (17.4)	18 (3.0)	126 (10.3)
Jaundice	44 (7.5)	21 (3.7)	65 (5.6)	40 (6.5)	34 (5.6)	74 (6.1)
Respiratory distress^c^	55 (9.4)	16 (2.8)	71 (6.1)	71 (11.5)	16 (2.7)	87 (7.1)
Severe anaemia^d^	33 (5.6)	9 (1.6)	42 (3.6)	67 (10.8)	26 (4.3)	93 (7.6)
Abnormal bleeding	8 (1.4)	19 (3.3)	27 (2.3)	3 (0.5)	20 (3.3)	23 (1.9)
Shock^e^	12 (2.0)	17 (3.0)	29 (2.5)	37 (6.0)	10 (1.7)	47 (3.8)
Haemoglobinuria^f^	8 (1.4)	14 (2.4)	22 (1.9)	9 (1.5)	16 (2.7)	25 (2.0)
Renal failure (oliguria or anuria)	4 (0.7)	8 (1.4)	12 (1.0)	3 (0.5)	4 (0.7)	7 (0.6)
Pulmonary oedema	1 (0.2)	2 (0.4)	3 (0.3)	6 (1.0)	3 (0.5)	9 (0.7)
Hypoglycaemia^g^	0	0	0	3 (0.5)	1 (0.2)	4 (0.3)
Any clinical or laboratory features	297 (50.5)	205 (35.7)	502 (43.2)	258 (57.7)	252 (41.7)	610 (49.8)
Malaria admission diagnoses						
“Malaria” (unclassified)	298 (50.7)	376 (65.5)	674 (58.0)	285 (46.0)	360 (59.6)	645 (52.7)
Any severe malaria diagnosis	195 (33.2)	175 (30.5)	370 (31.8)	229 (36.9)	179 (29.6)	408 (33.3)
Severe malaria	185 (31.5)	137 (23.8)	322 (27.7)	223 (36.0)	156 (25.8)	379 (31.0)
Cerebral malaria	13 (2.2)	31 (5.4)	44 (3.8)	5 (0.8)	17 (2.8)	22 (1.8)
Complicated malaria	6 (1.0)	7 (1.2)	13 (1.1)	5 (0.8)	11 (1.8)	16 (1.3)
Uncomplicated/non-severe	25 (4.3)	12 (2.1)	37 (3.2)	19 (3.1)	8 (1.3)	27 (2.2)
Any severe malaria criteria	380 (64.6)	298 (51.9)	678 (58.4)	433 (69.8)	336 (55.6)	769 (62.8)

^a^“Drowsiness, lethargy, confusion, unconsciousness, coma”, “AVPU < A” or “GCS < 15”

^b^“Unable to drink/breastfeed/sit/stand/walk” or “prostrated”

^c^“Acidotic/deep breathing”, “chest in-drawing” or “respiratory distress”

^d^“Hb < 5 g/dl” or “HCT < 15%”

^e^“Capillary refill ≥3 s”, “systolic BP < 80 mmHg in adults or < 50 mmHg in children” or “shock”

^f^“Dark urine” or “blood in urine”

^g^“Blood sugar < 2.2 mmol/l”

### Hospital readiness for implementation of the new case-management policy

The availability of artesunate increased by 21.3% between survey rounds (63.8–85.1%) while retrospective artesunate stock-outs declined by 27.5% (46.8–19.2%) (Table 2). The availability improved both at hospitals in low malaria risk areas (+ 26.5; 52.9 to 79.4%) and at those in high risk areas where, during the second round, none of the hospitals was found without injectable artesunate. Hospitals during both rounds less commonly stocked quinine (51% vs 49%). Despite significantly improved availability of artesunate, the second survey found 10.6% of hospitals (all from low risk areas) without any injectable anti-malarial in stock. The proportion of hospitals with at least one ward displaying an artesunate administration poster increased by 21.2% (51.1–72.3%) with posters commonly found in the paediatric (60%), less commonly in the female medical (32%) and the least commonly in male medical wards (23%). Yet only seven hospitals (14.9%) were found with all three wards having artesunate posters displayed. Finally, with respect to malaria diagnostics, microscopy was universally available during both rounds while malaria RDTs were stocked at less than a third of hospitals (31.9 and 29.8%, respectively) (Table 2).

**Table 2:** Hospital readiness characteristics, by survey round

N = 47	Round 1	Round 2
	n (%)	n (%)
Availability of injectable anti-malarials		
Artesunate in stock on survey day	30 (63.8)	40 (85.1)
Quinine in stock on survey day	24 (51.1)	23 (48.9)
Artemether in stock on survey day	6 (12.8)	2 (4.3)
Any injectable anti-malarial in stock on survey day	40 (85.1)	42 (89.4)
Expired artesunate in stock	12 (25.5)	1 (2.1)
Artesunate stock-out in past 3 months	22 (46.8)	9 (19.2)
Availability of malaria diagnostics		
Functional malaria microscopy	47 (100)	47 (100)
Malaria RDTs in stock	15 (31.9)	14 (29.8)
Artesunate administration poster in wards^a^		
Displayed in at least one ward	24 (51.1)	34 (72.3)
Displayed in all wards	6 (12.8)	7 (14.9)

^a^Paediatric, female and male medical ward

### Health worker readiness and their knowledge about artesunate policy and use

Table 3 shows health workers’ exposure to relevant trainings, guidelines and supportive supervision. No significant changes in the coverage of health workers trained on artesunate use has been observed (42.2% vs 40.7%; p = 0.764). A quarter (24.7%) of interviewed health workers have been trained through the NMCP malaria case-management training while lower coverage with a decline between rounds was observed in those exposed to artesunate CME sessions (17.4% vs 8.8%; p = 0.021). No significant differences were observed during the second round in the training coverage between clinicians and nurses (37.8% vs 44.1%; p = 0.371), paediatric and medical ward health workers (41.9% vs 39.3%; p = 0.723), and between high and low malaria risk areas (36.5% vs 42.3%; p = 0.503). Differences were observed between nurses and clinicians exposed to the NMCP malaria case-management training (30.6% vs 17.9%; p = 0.013). Finally, health workers’ exposure to any type of the supportive supervision during 3 months prior to the surveys increased from 16.2 to 26.4% (p = 0.042). Despite low levels of supervision on artesunate use during both rounds (8.7% v*s* 12.8%), clinicians compared to nurses were more commonly supported (23.8% vs 3.1%; p = 0.002), as were health workers in high risk areas compared to low risk areas (23.1% vs 8.6%; p = 0.019).

**Table 3:** Health workers’ coverage with support interventions, by survey round

Exposure to support activities	Round 1 N = 185	Round 2 N = 182	*p* value
	n (%)	n (%)	
Training exposure			
NMCP malaria case-management training			
Ever trained	51 (27.6)	52 (28.6)	0.848
Trained on artesunate use	42 (22.7)	45 (24.7)	0.670
Artesunate training through CMEs	32 (17.4)^a^	16 (8.8)	0.021
ETAT trained on artesunate use	20 (11.2)^a^	22 (12.1)	0.765
Any training on artesunate use	78 (42.2)	74 (40.7)	0.764
Guidelines exposure			
Malaria case-management guideline	52 (28.1)	66 (36.3)	0.143
Basic paediatric protocols	99 (53.8)^a^	109 (60.6)^a^	0.172
Supportive supervision			
Any supervisory visit in past 3 months	30 (16.2)	48 (26.4)	0.042
Supervision including severe malaria management	17 (9.2)	24 (13.2)	0.282
Supervision including artesunate use	16 (8.7)	23 (12.8)^a^	0.184

^a^Denominator excludes health workers without information available

Improvements in knowledge about artesunate treatment policy for severe malaria was observed (Table 4). The correctness of treatment responses increased from 73.5 to 85.7% (p = 0.002) for children and non-pregnant adults, from 43.2 to 53.9% (p = 0.085) for women in the first trimester of pregnancy, and from 52.4 to 63.7% (p = 0.067) for pregnant women in the second and third trimester. Quinine treatment was still the most common incorrect response (10.4% for children and non-pregnant adults, 22.5% in the second and third trimester, and 32.4% in the first trimester of pregnancy). The treatment knowledge for children and non-pregnant adults was higher among health workers exposed to artesunate training (91.9% vs 81.5%; p = 0.077). Similarly, artesunate trained health workers showed higher knowledge of recommended treatment for pregnant women in the second and third trimester (73.0% vs 57.4%; p = 0.039). Clinicians, compared to nurses, were more knowledgeable of artesunate treatment in the first trimester of pregnancy (61.9% vs 46.9%; p = 0.022). The knowledge of recommended artesunate dosing also significantly improved—from 42.7 to 64.6% (p < 0.001) for children less than 20 kg and from 70.3 to 80.8% (p = 0.052) in patients weighing over 20 kg (Table 4).

**Table 4:** Knowledge about treatment policies for severe malaria and artesunate use, by survey round

Health workers’ knowledge [correct responses in brackets]	Round 1 N = 185	Round 2 N = 182	p-value
	n (%)	n (%)	
Treatment policy for severe malaria			
Children and non-pregnant adults [AS]	136 (73.5)	156 (85.7)	0.002
First trimester of pregnancy [AS]	80 (43.2)	98 (53.9)	0.085
Second and third trimester of pregnancy [AS]	97 (52.4)	116 (63.7)	0.067
Follow on treatment [AL]	167 (90.3)	159 (87.4)	0.245
Artesunate preparation			
Preferred AS administration route [IV slow bolus]	140 (75.7)	149 (81.9)	0.110
AS reconstitution solution [bicarbonate]	141 (76.2)	152 (83.5)	0.100
AS dilution solution [saline or 5% dextrose]	138 (74.6)	135 (74.2)	0.932
Artesunate dosing for child < 20 kg			
Recommended [3 mg/kg]	79 (42.7)	117 (64.6)^a^	< 0.001
Below recommendation	75 (40.5)	41 (22.7)^a^	0.002
Above recommendation	3 (1.6)	6 (3.3)^a^	0.417
Doesn’t know	28 (15.1)	17 (9.4)^a^	0.105
Artesunate dosing for patient > 20 kg			
Recommended [2.4 mg/kg]	130 (70.3)	147 (80.8)	0.052
Below recommendation	3 (1.6)	0	0.084
Above recommendation	29 (15.7)	18 (9.9)	0.151
Doesn’t know	23 (12.4)	17 (9.3)	0.404
Artesunate treatment duration			
Minimum number of AS doses [3]	160 (86.5)	155 (85.2)	0.691
Time interval between first three doses [12 hourly]	84 (45.4)	114 (62.6)	0.001
Maximum days of AS if unable to take orally [7]	80 (43.2)	98 (53.9)	0.047

^a^Denominator excludes a health worker with missing information

### Malaria case-management practices

Of 1162 and 1224 suspected malaria admissions evaluated respectively during the first and the second survey rounds, a significant 7.7% improvement in the overall quality of malaria case-management was observed—from 48.6 to 56.3% (p = 0.004) (Table 5). The testing of suspected malaria patients was high during both rounds (88.6% vs 92.1%; p = 0.080) and nearly all patients at both rounds had malaria microscopy performed (99.0% vs 99.4%). A similar proportion of patients tested positive on admission (60.0% vs 61.4%) and only 5.2 and 8.1% of patients had at respective surveys malaria test repeated. Of 405 and 465 test positive patients with severe malaria, a significant 8.8% improvement was observed between rounds in the treatment practice with recommended parenteral artesunate—from 69.9 to 78.7% (p = 0.030) (Table 5). Nearly all (98%) artesunate treated patients were prescribed the IV route of administration. The use of parenteral quinine significantly declined between the survey rounds—from 24.9 to 14.0% (p = 0.004). Of 212 and 227 respectively evaluated test positive patients without severe malaria criteria, no significant changes in the treatment with recommended AL were observed. The levels of AL treatment were low during both survey rounds (8–9%). Most of these patients were treated similarly as confirmed severe cases with 72.7% treated with artesunate during the second round (Table 5).

**Table 5:** Malaria case-management practices, by survey round

	Round 1 (N = 1162)	Round 2 (N = 1224)	p-value
	n (%)	n (%)	
Composite performance	565 (48.6)	689 (56.3)	0.004
Malaria test done on admission	1, 029 (88.6)	1127 (92.1)	0.080
Malaria test repeated	53 (5.2)	91 (8.1)	0.034

Treatment for test positive severe cases	N = 405	N = 465	
Artesunate parenteral	283 (69.9)	366 (78.7)	0.030
Quinine parenteral	66 (16.3)	54 (11.6)	0.126
Artesunate and quinine parenteral	35 (8.6)	11 (2.4)	0.003
artemether–lumefantrine	16 (4.0)	26 (5.6)	0.245
Other anti-malarial treatments^a^	2 (0.5)	0	0.168
No anti-malarial treatment	3 (0.7)	8 (1.7)	0.322

Treatment for test positive non-severe cases	N = 212	N = 227	
artemether–lumefantrine	17 (8.0)	20 (8.8)	0.796
Artesunate parenteral	133 (62.7)	165 (72.7)	0.053
Quinine parenteral	40 (18.9)	32 (14.1)	0.316
Artesunate and quinine parenteral	16 (7.6)	3 (1.3)	0.006
Other anti-malarial treatments^b^	1 (0.5)	3 (1.3)	0.444
No anti-malarial treatment	5 (2.4)	4 (1.8)	0.672

Treatment for test negative severe cases	N = 208	N = 240	
No anti-malarial treatment	123 (59.1)	149 (62.1)	0.673
Artesunate parenteral	58 (27.9)	71 (29.6)	0.783
Quinine parenteral	14 (6.7)	6 (2.5)	0.128
Artesunate and quinine parenteral	5 (2.4)	1 (0.4)	0.120
artemether–lumefantrine	8 (3.8)	12 (5.0)	0.579
Other anti-malarial treatments^c^	0	1 (0.4)	0.328

Treatment for test negative non-severe cases	N = 204	N = 195	
No anti-malarial treatment	140 (68.6)	152 (78.0)	0.063
Artesunate parenteral	36 (17.7)	29 (14.9)	0.542
Quinine parenteral	16 (7.8)	7 (3.6)	0.157
Artesunate and quinine parenteral	3 (1.5)	0	0.091
artemether–lumefantrine	9 (4.4)	7 (3.6)	0.639

Treatment for not tested patients	N = 133	N = 97	
No anti-malarial treatment	54 (40.6)	29 (29.9)	0.110
Artesunate parenteral	45 (33.8)	58 (59.8)	0.002
Quinine parenteral	23 (17.3)	6 (6.2)	0.037
artemether–lumefantrine	10 (7.5)	3 (3.1)	0.147
Artesunate and quinine parenteral	1 (0.8)	1 (1.0)	0.829

^a^ Includes one artemether treatment and one artemether/quinine treatment

^b^Includes two DHA-PPQ, one artemether, and one artemether/quinine treatment

^c^Includes one artesunate/artemether treatment

Among test negative patients without severe malaria criteria improvements were also observed between the surveys (Table 5). Of 204 and 195 patients at respective rounds, a 9.4% improvements in adherence to guidelines were observed—from 68.6% not treated for malaria to 78.0% (p = 0.063). Of 208 and 240 test negative patients with severe malaria criteria, the changes in adherence to test negative results were not significant between rounds (59.1–62.1%; p = 0.673) and similar proportion of these patients remained treated with injectable artesunate (27.9% vs 29.6%). Among artesunate-treated test negative patients with severe malaria criteria only a minor increase in the recommended repeat testing was observed, from 3.4 to 8.5% (p = 0.238). Of six repeat blood slide tests in this patient group during the second round, only one test was positive and of the remaining five negative tests artesunate was discontinued for only one patient. Finally, in the smallest group of 133 and 97 patients who were not tested for malaria, and therefore not managed in accordance with guidelines, an increase in the use of artesunate was also observed between the survey rounds (from 33.8 to 59.8%; p = 0.002).

### Malaria case-management practices stratified by admission ward and risk area

**Fig. 3:**
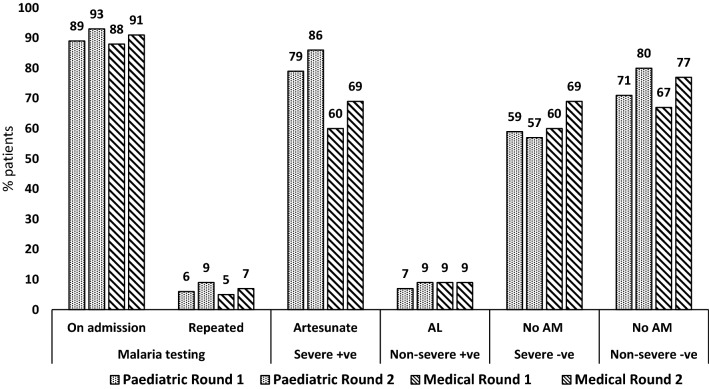
Key malaria case-management practices, by ward and survey round

**Fig. 4:**
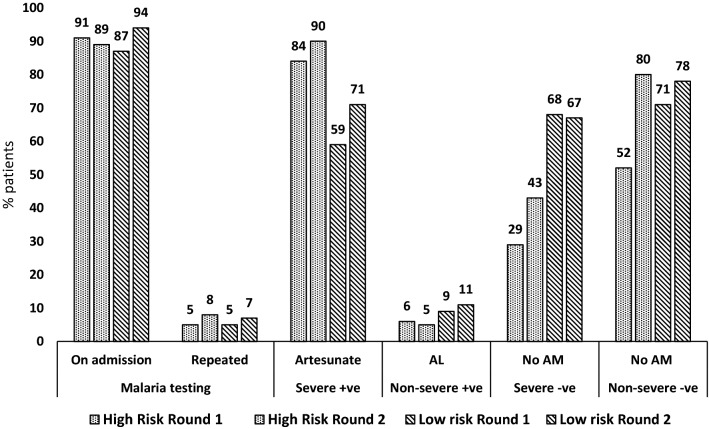
Key malaria case-management practices, by malaria risk and survey round

Several specifics of the case-management levels and trends were observed in the analyses stratified by admission ward and malaria risk area. First, composite performance showed similar improvements within each ward (+ 7.4; 54.1 to 61.5%; p = 0.019 among paediatric and + 8.0; 43.0 to 51.0%; p = 0.041 among adults) and within malaria risk area (+ 7.0; 51.1 to 58.1%; p = 0.024 in high risk and + 8.0, 47.5 to 56.0%; p = 0.029 in low risk areas). During the second round the composite performance was higher for paediatric patients compared to adults (61.5% vs 51.0%; p = 0.004) and without significant differences between high and low risk areas (58.1% vs 56.0%; p = 0.609). Second, artesunate treatment for confirmed severe cases increased within each ward (+ 7.1; 78.8 to 85.9%; p = 0.180 in the paediatric and + 9.3, 59.6 to 68.9%; p = 0.089 in the medical) and within risk areas (+ 6.7, 83.5 to 90.2%; p = 0.145 in high risk and + 11.9; 59.4 to 71.3%; p = 0.071 in the low risk area). In this category during the second round, children compared to adults (85.9% vs 68.9%; p = 0.001) and patients in the high risk compared to low risk areas (90.2% vs 71.3%; p = 0.002), were more commonly treated with artesunate. Third, adherence to “no anti-malarial” policy for test negative patients without severe criteria showed improvement trends across wards (+ 9.0; 70.5 to 79.5%; p = 0.210 in the paediatric and + 9.8; 67.0 to 76.8%; p = 0.177 in the medical) and areas of risk, particularly in the high risk (+ 28.3; 51.7 to 80.0%; p = 0.052), resulting in similar second round adherence levels between high and low risk areas (80.0% vs 77.7%; p = 0.849). Fourth, AL treatment of test positive patients without severe criteria remained low across all wards and risk areas (range 4.6–10.5%) and without changes within strata over time. Sixth, “no anti-malarial” practice for severe test negative patients increased over time only within the high risk areas (+ 13.9; 29.2 to 43.1%; p = 0.065), however remaining more common in low compared to high risk areas (67.2% vs 43.1%; p = 0.008). Finally, across both wards, risk areas and survey rounds testing of patients on admission for malaria was very high (range 87.5–93.5%) while repeat testing for malaria was very low (range 5.0–9.2%) (Figs. 3 and 4).

### Artesunate dosing practices

During both survey rounds weighing of patients was commonly performed for artesunate prescribed children in the paediatric ward (83.6 and 82.4% respectively) while it was rare among adults in medical wards (15.4 and 20.8% respectively). Yet recommended artesunate dosing of patients who had weight measured significantly improved between rounds—from 57.3 to 70.0% (p = 0.003) among all patients, from 54.9 to 65.6% (p = 0.049) for children < 20 kg requiring a dose of 3 mg/kg, and from 61.6 to 79.5% (p = 0.008) for patients > 20 kg requiring a dose of 2.4 mg/kg. Artesunate prescriptions below recommended dose also significantly declined—from 27.7 to 16.7% (p = 0.005) among all patients, from 35.9 to 22.1% (p = 0.008) for children < 20 kg, and from 13.4 to 4.3% (p = 0.017) for patients > 20 kg (Table 6).

**Table 6:** Correctness of artesunate dosing for weighed patients, by survey round

Artesunate dose	Children < 20 kg			Patients > 20 kg			All patients^a^		
	Round 1 N = 195	Round 2 N = 253	p-value	Round 1 N = 112	Round 2 N = 117	p-value	Round 1 N = 307	Round 2 N = 370	p-value
	n (%)	n (%)		n (%)	n (%)		n (%)	n (%)	
Recommended^b^	107 (54.9)	166 (65.6)	0.049	69 (61.6)	93 (79.5)	0.008	176 (57.3)	259 (70.0)	0.003
Below recommended	70 (35.9)	56 (22.1)	0.008	15 (13.4)	5 (4.3)	0.017	85 (27.7)	61 (16.7)	0.005
Above recommended	18 (9.2)	31 (12.3)	0.265	28 (25.0)	19 (16.2)	0.147	46 (15.0)	50 (13.5)	0.581

^a^31 patients without dose information were excluded

^b^3 mg/kg for patients < 20 kg; 2.4 mg/kg for patients > 20 kg

## Discussion

The capacity of Kenyan hospitals to provide parasitological malaria diagnosis is universal, continuous and reliant on malaria microscopy with uncommonly stocked RDTs. Despite an international interest in the use of RDTs across all levels of care [23], the results of this study are not surprising and they are in line with malaria diagnostic policy in Kenya promoting use of RDTs at lower level facilities where malaria microscopy is not available [24]. What however remains to be examined in Kenya are the levels reached with the quality assurance components of malaria microscopy at hospital laboratories—the activity shown to be feasible and successfully implemented in some areas of Kenya [25–28]. Major improvements in the availability of artesunate, decline in retrospective stock-outs, disappearance of expired stocks, and universal availability of artesunate in high malaria risk areas have also been observed during 2016. These encouraging trends should be however balanced against findings in low risk areas where 21% of hospitals had no artesunate and 15% were found without any injectable anti-malarial in stock. Artesunate stock-outs at hospitals have been rarely examined across Africa but they are unlikely to be unique to Kenya [29, 30]. While investigations of the supply chain are beyond the scope of this study, the interactions between survey teams and hospital pharmacists do suggest that broad range of the managerial issues related to the integration of commodity orders and delayed financial clearances of the supplies are likely factors constraining access to free artesunate. The future maintenance of the effective supply chain at hospitals with artesunate and resolved stock-outs at other hospitals will be critical for universal and continuous policy implementation. A significant increase in the coverage of hospitals with artesunate posters, a simple job aid facilitating its administration, has also been observed. Yet despite its distribution by study teams to all wards during the first survey, less than three-quarters of hospitals had at least one ward with displayed poster 6 months later. The results show that even simple interventions such as poster distributions require repeated engagements with hospitals to optimize the coverage.

The coverage of health workers exposed to any type of the training on artesunate use is still suboptimal (41%) and without an increase observed during 2016 despite 5000 health workers having been trained in this period. Only a quarter of inpatient health workers have been reached with the nationwide, annually undertaken, 3-day malaria case-management trainings—a substantial gap compared to two-thirds of outpatient health workers trained in Kenya through the same training [19] and mimicking low exposure levels reported among inpatient health workers in Uganda [9]. Careful selection of participants with greater inclusion of hospital health workers, and particularly clinicians as shown in this study, should be the future training priority. Single nationwide round of artesunate specific hospital CMEs undertaken in 2014/2015 increased number of trained health workers but not sufficiently to optimize the coverage. Further rounds of CME support should be reinstated while hospitals managers should ensure maximum health workers’ attendance. Finally, despite some increase in the exposure to supportive supervision, the coverage of only a quarter of supported health workers is low, especially among nurses, in low risk areas, and content-wise on appropriate artesunate use. These findings are in stark contrast with rural facilities where three quarters of outpatient health workers received supervisory visit in 2016 [19]. The reasons why county managers neglect easily accessible hospitals with supportive supervision visits should be further explored.

Health workers’ knowledge about artesunate treatment policy for severe malaria significantly improved. The awareness reaching 86% of health workers 4 years after the policy change is however not yet optimal and the major knowledge gaps remain for pregnant women, particularly in the first trimester (54%). These findings are in line with suboptimal levels of the treatment knowledge found for pregnant women with uncomplicated malaria in Kenya [31] as well as in other African countries [32]. The knowledge of recommended artesunate dosing improved between the rounds, both in children < 20 kg and patients over 20 kg. Despite these improvements, less than two-thirds of health workers knew about artesunate dosing of 3 mg/kg for children below 20 kg and these knowledge levels are significantly lower compared to patients over 20 kg where 2.4 mg/kg is recommended (81%). This pattern is likely due to more recent changes in WHO artesunate dosing recommendations for children < 20 kg from 2.4 to 3 mg/kg [33] whose implementation in Kenya started in 2015 and many health workers have not yet been updated. While relating dosing training messages with health workers’ knowledge was not possible in this study, higher awareness about artesunate treatment policy was observed among trained health workers and clinicians compared to nurses, the findings likely reflecting the training effects and prescribing roles of clinicians in the inpatient setting. It should be also acknowledged that the interactions between survey teams and health workers may have contributed to the improved knowledge.

The overall quality of inpatient malaria management significantly improved during the monitoring period. The test and treat practices for specific groups of admitted patients with suspected malaria revealed a series of strengths but also challenges in adherence to national guidelines. More positively, high testing rates (~ 90%) relying entirely on malaria microscopy show that, when health workers suspect malaria, the parasitological diagnosis is well ingrained into inpatient practices in Kenya. Similar testing levels at hospitals have been recently reported from smaller paediatric studies in Kenya [13, 15], Nigeria [16] and Uganda [11, 12], but not from all African countries [14]. In Kenya, earlier hospital reports [8] also found high testing rates suggesting longer term presence of this practice for inpatients and not necessarily a major shift following introduction of the universal malaria testing in 2010 [34, 35]. Perhaps most importantly, an increase in artesunate use for patients with confirmed severe malaria has been seen and relatively high levels (79%) of artesunate treatments have been reached. Supply chains for artesunate have been recently established and its hospital use on a larger scale has been rarely examined; however, of the few paediatric studies in high risk areas transition from quinine to artesunate have been observed [12, 15]. Artesunate treatment practices for adult patients and in low malaria risk areas have been scarcely reported [12] and our findings of significantly lower artesunate use in these patients highlight spatial and age priorities to be addressed. Finally, despite lagging behind the outpatient settings in Kenya [19], it was encouraging to observe improvements in no anti-malarial policy implementation for non-severe test negative patients, the practice among inpatients reaching relatively high adherence levels (78%) without age and malaria risk differences.

More negatively, while the recommended treatment for test positive non-severe patients is oral AL, no changes have been seen over time and the treatments were characterized by overuse of injectable anti-malarials, primarily artesunate. The practice of equalizing parenteral treatment with all malaria admissions has not only been a persistent problem in Kenya [15], but also reported in other African countries [14]. Such practice unnecessarily complicates administration of the treatment, increases cost to the health system and may further contribute to artesunate stock-outs. Related to the rational use of anti-malarials, 38% of severe test negative patients were treated for malaria, the rates within the range of 30–70% of malaria treated test negative admissions reported for various study populations across risk areas in Africa before and after 2010 policy change to universal malaria testing [10–12, 15]. This practice in Kenya, seems to be influenced by the background malaria prevalence and age-specific risks as evidenced by higher disregard of negative tests in high malaria risk areas and among paediatric patients. While initial treatment of severe test negative patents may be justified due to occasional sequestration of parasites and undetected parasitaemia, repeat testing with discontinuation of anti-malarial treatment after another negative test should be undertaken. Repeated testing is however uncommon in Kenya, as shown in this study and concurring with the reports of only 3% of repeated tests performed for admitted children with negative test result in Western Kenya [15]. Furthermore, lack of repeat testing compromises monitoring of treatment response for parasitaemic patients, currently recommended in Kenya at 12 h’ intervals during the first 3 days of admission [1]. The reasons behind the reliance upon malaria testing only on admission and lack of repeat testing should be further explored. Finally, with respect to artesunate dosing, no changes in the weighing practices for patients were observed and the large majority of adult patients were prescribed doses based on weight approximation. Yet, of those patients who had weight taken, improvements were observed in both weight groups (for patients < 20 and > 20 kg), the results mimicking the levels of improved knowledge about recommended dosing.

### Limitations

Several study limitations should be mentioned. First, data extractions based on the routine hospital records in resource limited settings in Africa are inevitably subject to documentation biases, especially with respect to the documentation of clinical signs and symptoms which are necessary to identify suspected severe malaria cases according to guidelines [13, 36]. Therefore, this study approach based on health workers’ suspicion of malaria provides information about case-management practices from health workers’ perspective of malaria suspicion but not in relation to the unknown universe of true suspected malaria cases. The results are however comparable with other studies in Kenya which followed similar analytic approaches [13, 15]. Second, the incompleteness of hospital registers used to select patient files has been a constraint precluding counts of the study defined universe of suspected malaria patients and subsequent weighted analyses based on the probability of selection. Third, the correctness of dosing practices refers to those patients with documented weight but not to all artesunate prescribed patients. Fourth, the results of these surveys apply to county referral hospitals and not to smaller facilities with inpatient capacities where readiness and practices might be different but where higher possibility of suboptimal medical filing systems may further exacerbate data collection limitations. Finally, multiple comparison tests undertaken through exploratory analyses may have also resulted in some of the results being significant by chance.

## Conclusions

The findings of inpatient surveys at county referral hospitals revealed that majority of key health systems and malaria case-management indicators focusing on the translation of artesunate treatment policy for severe malaria into practice showed improvements in 2016. Increased availability of artesunate, greater awareness and knowledge of health workers about new treatment policy, but also interactions of the study teams with hospital health workers, have likely contributed to these trends. Despite improvements, gaps do remain in several health systems and case-management areas which are often specific to different inpatient populations and malaria risk areas. The quantity and the quality of ongoing health systems interventions accompanied with close monitoring will ultimately determine the success of the policy translation.
